# Generation of a Retinoblastoma (*Rb)1*-inducible dominant-negative (DN) mouse model

**DOI:** 10.3389/fncel.2015.00052

**Published:** 2015-02-23

**Authors:** Shikha Tarang, Songila M. S. R. Doi, Channabasavaiah B. Gurumurthy, Donald Harms, Rolen Quadros, Sonia M. Rocha-Sanchez

**Affiliations:** ^1^Department of Oral Biology, Creighton University School of DentistryOmaha, NE,USA; ^2^Mouse Genome Engineering Core Facility, Department of Genetics Cell Biology and Anatomy, University of Nebraska Medical CenterOmaha, NE, USA

**Keywords:** retinoblastoma, pre-procathepsin B, dominant-negative, doxycycline, inner ear, hair cell, regeneration

## Abstract

Retinoblastoma 1 (*Rb1*) is an essential gene regulating cellular proliferation, differentiation, and homeostasis. To exert these functions, *Rb1* is recruited and physically interacts with a growing variety of signaling pathways. While *Rb1* does not appear to be ubiquitously expressed, its expression has been confirmed in a variety of hematopoietic and neuronal-derived cells, including the inner ear hair cells (HCs). Studies in transgenic mice demonstrate that complete germline or conditional *Rb1* deletion leads to abnormal cell proliferation, followed by massive apoptosis; making it difficult to fully address *Rb1*’s biochemical activities. To overcome these limitations, we developed a tetracycline-inducible *TetO-CB-myc6-Rb1* (CBRb) mouse model to achieve transient and inducible dominant-negative (DN) inhibition of the endogenous RB1 protein. Our strategy involved fusing the *Rb1* gene to the lysosomal protease pre-procathepsin B (CB), thus allowing for further routing of the DN-CBRb fusion protein and its interacting complexes for proteolytic degradation. Moreover, reversibility of the system is achieved upon suppression of doxycycline (Dox) administration. Preliminary characterization of DN-CBRb mice bred to a ubiquitous rtTA mouse line demonstrated a significant inhibition of the endogenous RB1 protein in the inner ear and in a number of other organs where RB1 is expressed. Examination of the postnatal (P) DN-CBRb mice inner ear at P10 and P28 showed the presence of supernumerary inner HCs (IHCs) in the lower turns of the cochleae, which corresponds to the described expression domain of the endogenous *Rb1* gene. Selective and reversible suppression of gene expression is both an experimental tool for defining function and a potential means to medical therapy. Given the limitations associated with *Rb1*-null mice lethality, this model provides a valuable resource for understanding RB1 activity, relative contribution to HC regeneration and its potential therapeutic application.

## INTRODUCTION

Retinoblastoma 1 is the founding member of the pocket proteins family, which also includes p107/*Rbl1* and p130/*Rbl2* genes (Cobrinik, 2005). *Rb1* has well-established roles in a wide variety of tissues for the regulation of cell proliferation, differentiation, and apoptosis through interactions with a growing number of molecules, including the E2F family of transcription factors that regulates the cell cycle (Korenjak and Brehm, 2005; Sun et al., 2006). Association of un- or hypophosphorylated “active” RB1 with different members of the E2F family prevents entry into the S phase of the cell cycle (Korenjak and Brehm, 2005; Sun et al., 2006). During the G1 phase of a normal cell cycle, RB1 is progressively phosphorylated by the complex formed by cyclin D1 and members of the cyclin D-dependent kinases (CDKs; Adams, 2001). Phosphorylated RB1 becomes “inactive,” releasing its associated E2F transcription factor, thereby allowing for transition into the S phase (Korenjak and Brehm, 2005; Sun et al., 2006). For more than two decades, it has been known that inactivation of the *Rb1* pathway is a common feature in virtually all human tumors (Sherr, 1996; Classon and Harlow, 2002; Sherr and McCormick, 2002). Such noteworthy findings suggest that it is nearly impossible for a human cell to undergo proliferation without inactivating *Rb1* (Sherr, 1996). Nevertheless, most of the mechanisms underlying *Rb1* activity in quiescent and proliferating cells remain to be addressed.

Consistent with RB1’s nodal role in multiple pathways, experimental attempts to conventionally delete RB1 in transgenic mice have led to abnormalities in the hematopoietic and nervous system (Lee et al., 1992), as well as in bones (Thomas et al., 2001), kidneys (Zhu et al., 2009), teeth (Andreeva et al., 2012), skin (Wang et al., 2014), the digestive tract (Guo et al., 2009), cochlea (Mantela et al., 2005; Weber et al., 2008), and retina (Knudson, 1971; Lohmann and Gallie, 2004), followed by massive cell death and embryonic lethality at midgestation (Lee et al., 1992; Wu et al., 2003). While conditional *Rb1* deletion through the Cre-Lox recombination system helps overcome problems with early embryonic lethality, it still leads to massive cell death, as expected from the permanent deletion of such a key cell survival and homeostasis regulator (Chau and Wang, 2003; Mantela et al., 2005; Weber et al., 2008).

During the past decade, there has been a growing interest in exploring the potential therapeutic application of *Rb1* inactivation in tissue regeneration (Bakay et al., 2006; Goodrich, 2006; Du and Searle, 2009; Knudsen and Wang, 2010; Wang et al., 2013) and, in particular, in HC regeneration (Mantela et al., 2005; Sage et al., 2005, 2006; Weber et al., 2008). Nevertheless, to this date there are no models available that would allow for reversible inactivation of *Rb1* and its associated factors. Such studies would be greatly facilitated by using mice harboring *Rb1* conditional null alleles. We report here the generation and characterization of the *TetO-DN-CB-myc6-Rb1* (DN-CBRb) mouse model, which combines the inducible nature of the tetracycline-controlled transcriptional activation (TetO) system, the lysosomal fusion protease pre-procathepsin B (CB), and part of the *Rb1* coding sequence to generate a dominant-negative (DN) mutant RB1. As with any other protein destined to the lysosome, CB is synthesized by endoplasmic reticulum (ER)-bound ribosomes, post-translationally modified in the Golgi, and eventually routed to the lysosome (Kominami et al., 1991; Li et al., 1996). On the other hand, RB1 physically interacts with and modulates the activity of many different cellular proteins (Morris and Dyson, 2001; Goodrich, 2006). Hence, the combination of the fusion protease CB with the *Rb1* gene results in a transgene that can exert a DN effect upon the endogenous RB1 protein, as well as any other protein that associates with RB1 (Li et al., 1996, 2000). DN mutations are most easily described in proteins that function as dimers or multimers. To date, there is no evidence of RB1 homodimerization. Nonetheless, the inherent nature of its activity allows for the presence of multiple molecules associated with RB1 at any given time (Goodrich, 2006). Whenever the RB1-interacting complex has more than one RB1 binding site, which is occupied by a DN-CBRb protein, it will elicit DN inhibition of the endogenous RB1 protein and result on an RB1-null phenotype. Moreover, if at least one RB1 molecule in the RB1 interacting complex is a DN-CBRb, it will prevent the entire complex from fulfilling its normal role, diverting it to the lysosome for degradation.

We describe here the generation of a DN RB1 transgene and provide evidence of its functionality *in vitro* and *in vivo*. Breeding of this mouse to tissue-specific promoters driving either tTA or rtTA lines, in the absence or presence of doxycycline (Dox), respectively, will result in the expression of the DN-CBRb fusion protein, which can bind to RB1-interacting molecules, promoting its lysosomal degradation. Initial characterization of the DN-CBRb mouse model supports DN inhibition of the RB1 protein in a number of different systems where *Rb1* expression is known, including the inner ear. Consistent with RB1’s expression profile in the postnatal (P) organ of Corti (OC; Mantela et al., 2005), supernumerary inner HCs (IHCs) were observed along the length of the cochlea at P10 and P28, particularly concentrated in the middle and basal turns of the cochlea. The intrinsic property of the CB fusion protein, associated with RB1-mediated protein–protein interaction and combined with the reversible inducibility of the TetO system, makes this an interesting mouse model to assess the potential for transient and reversible *Rb1* inactivation to regenerate lost HCs.

## MATERIALS AND METHODS

### ANIMALS

The DN-CBRb transgenic developed in this study is viable and exhibits no developmental abnormalities. Genomic DNA from wild-type and DN-CBRb positive (DN-CBRb^+^) mice were submitted to standard PCR as described below. PCR products from both genotypes were purified and sequenced using an ABI Prism® 3100 genetic analyzer to confirm the mutation site. The rtTA (B6.Cg-Gt(ROSA)26Sortm1(rtTA*M2)Jae/J: Jaxmice stock number 006965) mouse line was purchased from the Jackson Laboratories. Double positive mice carrying both the DN-CBRb transgene and rtTA inducer allele (DN-CBRb^+^/*ROSA-CAG-rtTA*^+^) were used for experiments. Experimental negative control groups consisted of mice negative for CBRb and positive for rtTA (DN-CBRb^-^/*ROSA-CAG-rtTA*^+^), as well as mice positive for CBRb and negative for rtTA (DN-CBRb^+^/*ROSA-CAG-rtTA^-^*). Generation of the DN-CBRb transgenic line and all animal care and experiments associated with this study were approved by the Creighton University Institutional Animal Care and Use Committee (IACUC).

### GENERATION OF *CB-Myc_6_-Rb1* TRANSGENIC CONSTRUCT

**FIGURE 1:**
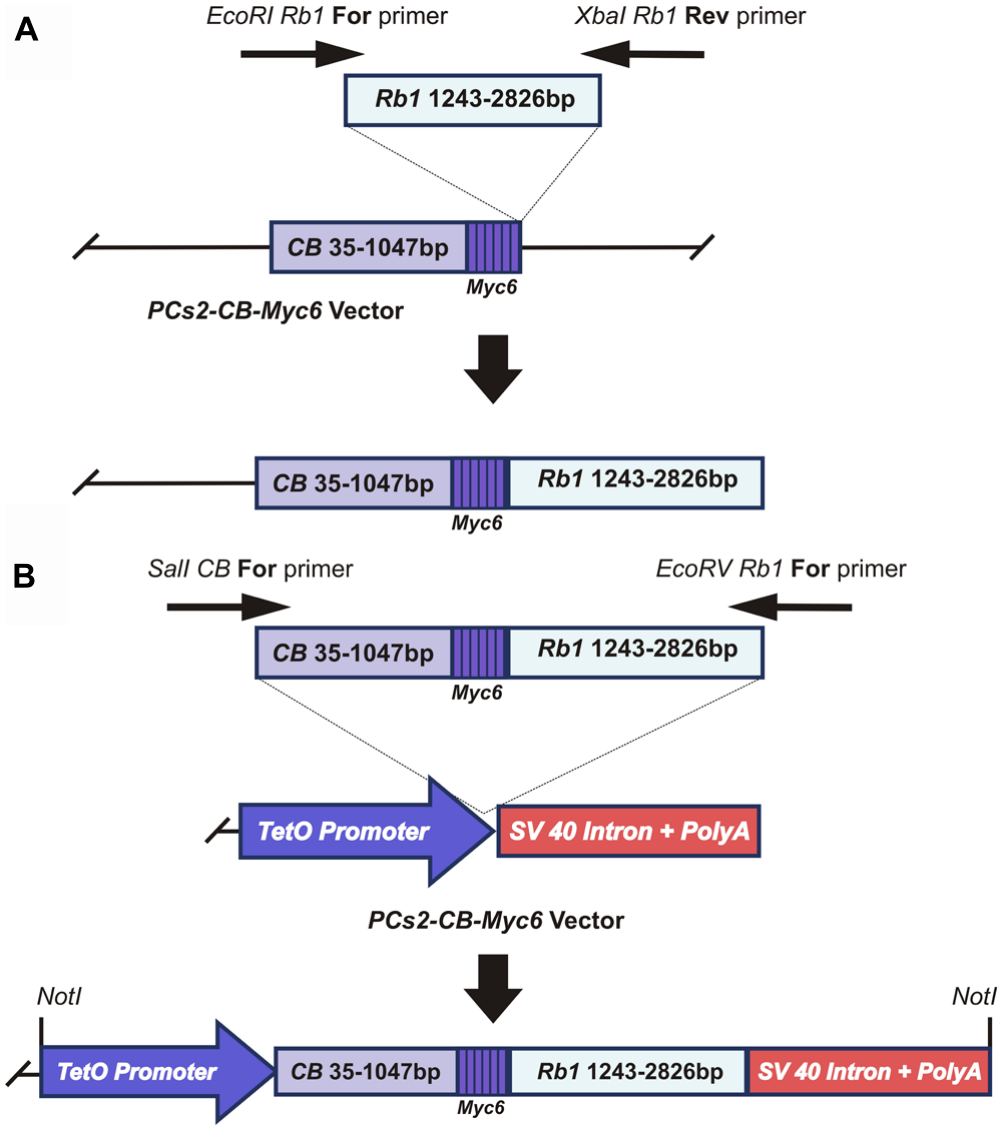
**Generation of the *CB-Myc6-Rb1* construct. (A)** cloning of the *Rb1* fragment into the *pCS2-CB-Myc6* vector. A 1583bp *Rb1* gene product was PCR amplified using Forward and Reverse primers. The resulting *Rb1* fragment was cloned between *EcoRI* and *Xbal* restriction sites of pCS2 vector containing *CB-Myc6* construct (a 1012bp *CB* gene fragment and a hexameric *Myc6* sequence tag), as described previously (Li et al., 1996, 2000). **(B)** The *CB-Myc6-Rb1* fragment was cloned into the *pTetSplice* vector between the *SalI* and *EcoRV* restriction sites.

To test the possibility of using fusion with a lysosomal protease as an effective means of causing DN inhibition of RB1, we amplified a 1583bp-long *Rb1* cDNA fragment corresponding to the amino acid region 369–896 of the RB1 protein (528 amino acids). This fragment was cloned between the *EcoR1* and *XbaI* restriction sites of the pCS2+CB-Myc_6_ vector (a gift from Dr. Marshal Horwitz, University of Washington; Li et al., 1996, 2000), to fuse it with the 1012bp long CB-Myc_6_ construct (337 amino acids; **Figure 1A**). The fusion product resulted in a peptide of 865 amino acids with CB- Myc_6_ at the N-terminus and *Rb1* at the C-terminus. The fusion product has an approximate size of 108 kDa, which is slightly smaller than the endogenous RB1 protein (∼110 kDa). The CB- Myc_6_-RB fusion fragment was PCR amplified and cloned into a pTet-splice vector between the *SalI* and *EcoRV* restriction sites to obtain the Tet(O)-DN-CBRB under tetracycline promoter (**Figure 1B**).

### *IN VITRO* TESTING OF THE DN-CBRb TRANSGENE AND GENERATION OF THE TRANSGENIC MOUSE

**FIGURE 2:**
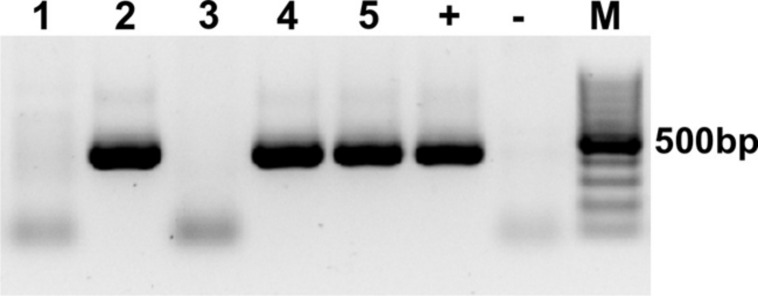
**Genotyping of the DN-CBRb mouse model.** PCR with primers specific for the *CB-Rb1* fusion transgene resulted in a 401 bp band in the DN-CBRb-positive animals (lanes 2, 4, and 5), but not in those lacking the transgene (lanes 1 and 3). rtTA genotype was carried following the protocol available at the Jackson Laboratories website (http://jaxmice.jax.org/strain/006965.html). (+) = Control DNA showing the transgene. (-) = Control PCR not containing DNA. *M* = 100 bp DNA ladder.

To stimulate *in vitro* overexpression and test the reversibility of the transgene, purified pTet-Splice plasmid containing the DN-CBRb cassette was co-transfected into HEK293 cells, with both DN-CBRb and the tetracycline-controlled transactivator pCMV-Tet3G vector (Clonetech Laboratories, Inc.), for 24 h. After that period, Dox media was removed and a subset of cells were washed from two to three times with PBS and incubated in Dox-free media for an additional 24 h to allow for inactivation of the transgene. Control samples consisted of co-transfected cells not treated with Dox, as well as HEK293 cells transfected only with the transactivator pCMV-Tet3G vector and treated with Dox for a 24-h period. Upon confirmation of effective Dox regulation of the DN-CBRb transgene, the entire cassette from the promoter up to the end of the SV40 polyA terminator element was excised out of the vector, gel purified, and used for the generation of the transgenic founder mice. The founder mice were generated using standard techniques at the Mouse Genome Engineering Core facility, University of Nebraska Medical Center. Pups were genotyped using a primer set specific to the *CB-Rb1* fusion region, consisting of forward primer 5′ CTGTGGCATTGAATCAGAAATTGTGGCTGG 3′ and reverse primer 5′ GTACTTCTGCTATATGTGGCCATTACAACC 3′ (**Figure 1B**), which amplified a product approximately 401 bp (60 bp CB region + 341 *Rb1* region; **Figure 2**). Several independent DN-CBRb founder lines were identified. Five of these lines were confirmed for germline transmission of the transgene. Two of those lines were further bred to establish breeding colonies and to test the transgene expression (i.e., lines 7 and 14). However, the results herein presented correspond to data collected from line 14 only. Reproductive problems with line 7 have precluded us from fully analyzing that line.

### TRANSFECTION OF THE DN-CBRb TRANSGENE IN HEI-OC1 CELLS

HEI-OC1 cells, kindly provided by Dr. Fedrico Kalinec (David Geffen School of Medicine, UCLA, Los Angeles, CA, USA), were maintained at 33°C with 10% CO2 (at permissive conditions) with 10% DMEM (#11965-084, GIBCO-BRL). 10,000 cells were plated on a 96-well plate and allowed to incubate overnight. On the following day transient transfection of DN-CBRB and rtTA construct was done using lipid based transfection reagent DharmaFECT Duo (T-2010-03). HEI-OC1 transfection rate was calculated based with pmR-ZsGreen1 (Clontech), wherein the transfected cells exhibited green fluorescence. 2 ug of pmR-ZsGreen1 was transfected in parallel, in a separate well. 1 ug/mL Dox was provided for experimental samples. Plasmid expression was measured 24 h after transfection and transfection rate was calculated by counting the number of GFP positive cells over the total cells labeled with Hoechst. A transfection rate of ∼70% was estimated. Of note, the treatment of untransfected HEI-OC1 cells with Dox did not affect RB1 protein expression or cell proliferation. Cell proliferation was assessed by CyQuant NF cell proliferation kit (Invitrogen, C35007). Cell proliferation assay was performed 48 h after transfection, according to manufacturer’s protocol. Briefly, the cell culture media was removed and 100 μl of 1X dye binding solution was added into each microplate well. The microplate was incubated for additional 1 h at 37°C. The fluorescence intensity for each sample was measured using fluorescence microplate reader (FLUOstar OPTIMA, BMG Labtech), with excitation at 485 nm and emission detection at 530 nm. To quantify any potential cell death following transfection, HEI-OC1 cells were trypsinized and counted. 0.1 × 10^6^ cells were plated on a coverglass in a 12-well plate and incubated overnight at 37°C. On the following day, cells were transfected with the DN-CBRb and rtTA constructs using DharmaFECT Duo (T-2010-03), lipid based transfection reagent. Positive control samples consisted of untransfected HEI-OC1 cells treated with 1 μM Staurosporine for 4 h. After 48 h of incubation, HEI-OC1 cells were analyzed for detection of active caspases according to the manfacturer’s protocol (Millipore, APT523). Briefly, 1X CaspaTag reagent solution was added and the cells were incubated at 37°C for 1 h. 5 ug/ml DAPI was used to label the nuclei of the dying cells for 10 min at room temperature. The cells were washed twice with 1X wash buffer and fixed using the supplier provided fixative. Thereafter, the coverslips were mounted and observed under the epifluorescence microscope (Nikon Eclipse 80i).

### *IN SITU* HYBRIDIZATION (ISH)

Whole-mount ISH detecting DN-CBRb mRNA was performed on Dox-treated (experimental) and untreated (control) P21 DN-CBRb^+^/*ROSA-CAG-rtTA*^+^ mice cochleae as previously described (Barritt et al., 2012). An approximately 600 nucleotides long riboprobe was generated by *in vitro* transcription of PCR products derived from DN-CBRb cDNA using primer 5′GGCTGGCAGCCAACTCTTGGAACCTTGACTGG. Reacted tissues were whole mounted in glycerol to be observed under the microscope or embedded on OTC media prior to frozen sectioning on a microtome cryostat. Whole mount and sections were imaged by differential interference contrast microscopy on a Nikon Eclipse 80i microscope. Sections of the cochlea and spiral ganglion neurons were counterstained with Nuclear Fast Red (Vector Laboratories, H3403).

### RNA EXTRACTION AND QUANTITATIVE RT-PCR ANALYSIS

Total RNA was isolated from dissected cochleae using RNeasy kit (Qiagen) according to the of the manufacturer’s instructions. Up to 20 μg of total RNA were treated with RNase-free DNase (Turbo DNfree; Ambion), and RNA concentration and quality were assessed on a Nanodrop spectrophotometer and Agilent Bioanalyzer, respectively. 250 ng of RNA were reverse transcribed using MultiScribe reverse transcriptase and random primers (Applied Biosystems). Semi-quantitative PCR assay was done in triplicate for each sample using SYBR green (Applied Biosystems, StepOne plus system). A corresponding amplification for each sample was performed with template without reverse transcriptase enzyme and used as control to rule out any possible genomic contamination. Amplification was performed using primers set specific to the CB-Rb1 fusion region, consisting of forward primer: 5′ CTGTGGCATTGAATCAGAAATTGTGGCTGG-3′ and reverse primer 5′TACTTCTGCTATATGTGGCCATTACAACC-3′, for 40 cycles. The relative quantitation of mRNA abundance was normalized to endogenous β-Actin using StepOne Software (Applied Biosystems; version 2.1). *T*-tests were performed on the normalized gene expression values to determine whether differences were statistically significant. *P* < 0.05 was considered significant.

### REGULATION OF THE DN-CBRb TRANSGENE *IN VIVO*

To enable transgene expression, the DN-CBRb mice were bred to the *ROSA-CAG-rtTA* tetracycline inducer line. Genotyping of the rtTA construct was performed as described at the Jackson Laboratories website (http://jaxmice.jax.org/strain/006965.html). Double-positive (DN-CBRb^+^/*ROSA-CAG-rtTA*^+^) and negative controls (DN-CBRb^-^/*ROSA-CAG-rtTA*^+^, DN-CBRb^+^/*ROSA-CAG-rtTA^-^*) mice were treated with 2 mg/ml Dox in drinking water (post-weaning) or through their lactating mothers (pre-weaning). Alternative Dox doses have been tested (e.g., lower or higher than 2 mg/ml). However, as the method relies on the volume of water consumed by the animals, lower doses proved to be less efficient, resulting on a high variability on the phenotype. Such variability was attributed to a decrease on the volume of water consumed by some animals, and consequently smaller Dox absorption. When higher than 2 mg/ml was used, animals tended to drink less water (and absorb less Dox) due to Dox’s bitter taste. Further experiments with injectable Dox at concentrations higher than 2 mg/mL showed no differences from what we normally observe when providing 2 mg/mL Dox in the drinking water. Hence, we proceeded with Dox in drinking water. At the end of the Dox-treatment, several tissues were harvested (e.g., cochlea, eye, liver, heart, lung, and kidney) from DN-CBRb^+^/*ROSA-CAG-rtTA*^+^ and DN-CBRb^+^/*ROSA-CAG-rtTA^-^* control mice and either stored in RNAlater® stabilization solution (Ambion) for subsequent RNA or protein extraction or kept in 4% paraformaldehyde (PFA) for histological analysis.

### WESTERN BLOT

The tissues collected in RNA later and stored at -80°C were homogenized in Ripa lysis buffer (Thermo scientific #89901) with protease inhibitor (Thermo scientific #88664). Homogenization was done using Omni Homogenizers. Lysates obtained from tissues or cells were centrifuged at 14,000 rpm for 20 min at 4°C. The supernatant was used for protein estimation using the Lowrey method (BioRad DC protein assay kit #500–0112). Thereafter, 20 μg of protein was resolved on 10% SDS-PAGE and proteins were then transferred to PVDF membranes in a Bio-Rad TransBlot apparatus according to the manufacturer’s instructions at 100 V for 90 min. The proteins were transferred to PVDF membrane (Millipore Immobilon-P IPVH304FO) at 100 V for 90 min using a Biorad wet-tank apparatus. After incubation in blocking solution, the PVDF membrane was blocked in 5% blocking solution for 2 h at room temperature and probed overnight at 4°C with anti-RB1 (antibody against total RB1 protein; Abcam, ab6075), anti- active caspase 3 (Millipore, AB3623), anti -c-myc (Sigma, c3956) and anti β-actin (Santa Cruz, sc-69879) overnight at 4°C. The membranes were then washed and incubated in appropriate HRP-conjugated secondary antibodies, anti-rabbit (Santa Cruz, sc-2054) and anti-mouse (Santa Cruz, sc-358923) for 1 h at RT. After washing, peroxidase-bound protein bands were visualized by chemiluminescence using ECL substrate (Pierce, Rockford, IL, USA).

### HISTOLOGICAL ANALYSIS

Whole mount immunohistochemistry was performed as previously described (Rocha-Sanchez et al., 2011). Immunohistological staining of Myosin VIIa (M7a; Proteus Biosciences) and active capsase 3 (Millipore, AB3623) was performed on DN-CBRb^+^/*ROSA-CAG-rtTA*^+^ and DN-CBRb^-^/*ROSA-CAG-rtTA*^+^ formalin-fixed whole-mount preparations of dissected cochlear neurosensory epithelia. Tissue pieces (apex, middle, and base) were block/permeabilized with 5% NGS/0.1% Tween 20 at room temperature for 2–3 h, incubated with a primary antibody for 48 h in blocking buffer, and washed three times with PBS. Samples were then labeled with Alexa 568-conjugated secondary antibodies (1:500; Invitrogen), washed with PBS, counterstained with DAPI (5 ug/ml) for 2 h at room temperature. The cochleae were then mounted using Prolong anti-fade (Invitrogen) and imaged using a Leica TCS SP8 MP confocal microscope. Cell counting was performed on 200X images obtained from three different regions (apex, middle, and base) of the DN-CBRb^+^/*ROSA-CAG-rtTA*^+^ and DN-CBRb^-^/*ROSA-CAG-rtTA*^+^ (control) cochleae. Statistical analysis between the two groups was done using paired *t*-test. Values are represented as ±SEM. *P* < 0.05 was considered significant.

### PHALLOIDIN LABELING

F-actin was stained with Alexa 488 conjugated phalloidin. Briefly, formalin-fixed cochleae were washed with PBS three times and permeabilized with 0.25% Triton X-100 for 10 min. Thereafter, the cochlear tissue was labeled with Alexa 488-phalloidin (1: 200; Sigma–Aldrich) for 30 min. After three washes with PBS, the specimens were counterstained with DAPI, and imaged using Leica TCS SP8 MP confocal microscope.

### PROLIFERATION ASSAY

To label mitotically active cells, a single, subcutaneous injection of the thymidine analog 5-ethynyl-2′-deoxyuridine (EdU; 50 mg/kg) in DMSO was administered to DN-CBRb^+^/*ROSA-CAG-rtTA*^+^ and DN-CBRb^+^/*ROSA-CAG-rtTA^-^* mice 4 h before tissue harvesting. EdU incorporation into DNA of whole-mount cochlea was detected using the Click-iT EdU Alexa 488 Fluor Imaging kit (Invitrogen) and double stained with DAPI following the manufacturer’s instructions and experimental procedures previously described (Kaiser et al., 2009; Rocha-Sanchez et al., 2011). Samples were imaged using a Leica TCS SP8 MP confocal microscope.

## RESULTS

### GENERATION OF THE TEMPORALLY AND CONDITIONALLY INDUCIBLE DN-CBRb TRANSGENE

To overcome the inherent problems associated with complete and permanent *Rb1* deletion in mice, we sought to develop a DN version of the RB1 protein that would not only inhibit RB1 activity, regardless of the presence of functional endogenous RB1, but also bind to and trigger proteasome-mediated degradation of the protein complexes that normally associate with RB1. To this end, we modified a previously published system (Li et al., 1996, 2000) consisting of the fusion of the preprocathepsin B protease associated with most of the entire pocket domain and C-terminal region of *Rb1*. The resultant CB-myc6-RBf1ΔC (DN-CBRb) cassette has a carboxyl-terminal deletion between residues 369 and 896 (Li et al., 1996, 2000). To avoid the complications associated with constitutive *Rb1* elimination, the DN-CBRb cassette was cloned under the tetracycline inducer Tet(O) promoter as described in materials and methods (**Figures 1A,B**). *In vitro* testing of the construct was performed in OC derived HEI-OC1 cells by transient transfection. Expression of the fusion protein was detected by Western blot with anti-RB1 antibody, as described below.

### *IN VITRO* ASSESSMENT OF THE *CB-Myc_6_-Rb1* CONSTRUCT

**FIGURE 3:**
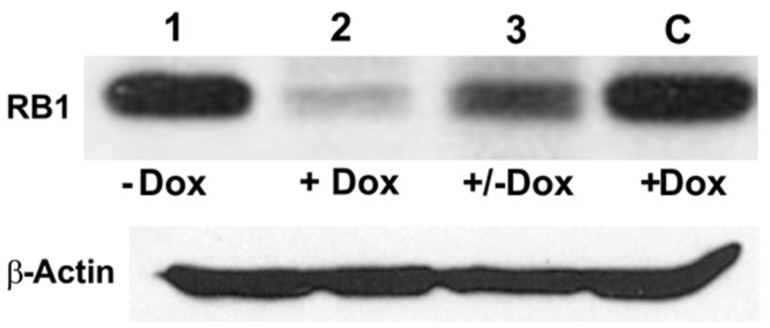
**TetO-driven, Dox-activated expression of the DN-CBRb transgene leads to downregulation of the endogenous RB1 *in vitro*.** HEK293 cells, which endogenously express RB1 (Chano et al., 2006), were co-transfected with *TetO-DN-CB-myc_*6*_-Rb1,* and the pCMV-Tet3G transactivator vector. In the absence of Dox, co-transfected HEK293 cells stably expressed RB1 (lane 1). Addition of Dox to the system for a 24-h period led to *TetO-DN-CB-myc_*6*_-Rb1* activation and RB1 downregulation (lane 2). Culturing a subset of those Dox-treated cells in a Dox-free media for an additional 24 h allowed for RB1 expression to be resumed (lane 3). C = Control HEK293 cells transfected with pCMV-Tet3G only, treated with Dox.

To test the effectiveness of Dox-regulated DN-CBRb activation and the reversibility of the system, HEK 293 cells were co-transfected with purified pTet-Splice plasmid containing the *CB-Myc_6_-Rb1* construct and the pCMV-Tet3G vector. Addition of Dox to the culture media significantly inhibited the expression of the endogenous RB1 protein (**Figure 3**). Following a 24-h period, Dox treatment was withdrawn and a subset of those Dox-treated HEK 293 cells was provided with a Dox-free media for an additional 24 h. Consistent with the reversible nature of the system, RB1 expression was restored in those cells after Dox removal from the culture media (**Figure 3**). Noteworthy, no changes in RB1 expression were observed on co-transfected HEK 293 cells not treated with Dox or on Dox-treated HEK 293 cells transfected with the pCMV-Tet3G vector only (**Figure 3**).

**FIGURE 4:**
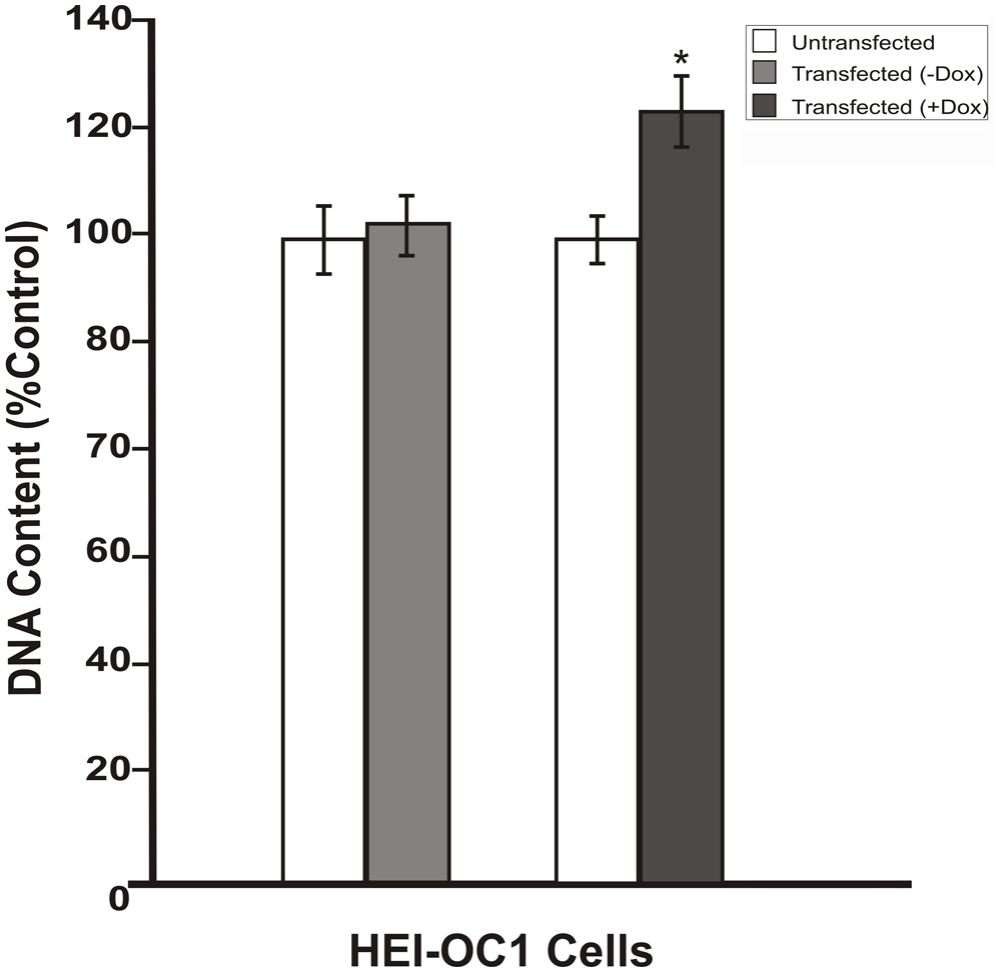
**Dox-mediated DN-CBRb transgene activation increases cell proliferation.** HEI-OC1 cells co-transfected with purified pTet-Splice-DN-CBRB and the pCMV-Tet3G vectors were cultured in the absence (-) or presence (+) of Dox. Cell proliferation was determined 48 h after transfection using CYQUANT NF Proliferation Assay. Percentage change in proliferation was calculated using change in fluorescence values with untransfected cells as control. A modest, but significant increase in cell number was observed on transfected cells following Dox treatment. In contrast, no significant changes were observed between transfected HEI-OC1 cells not treated with (-Dox) and the untransfected control. *P < 0.05.

**FIGURE 5:**
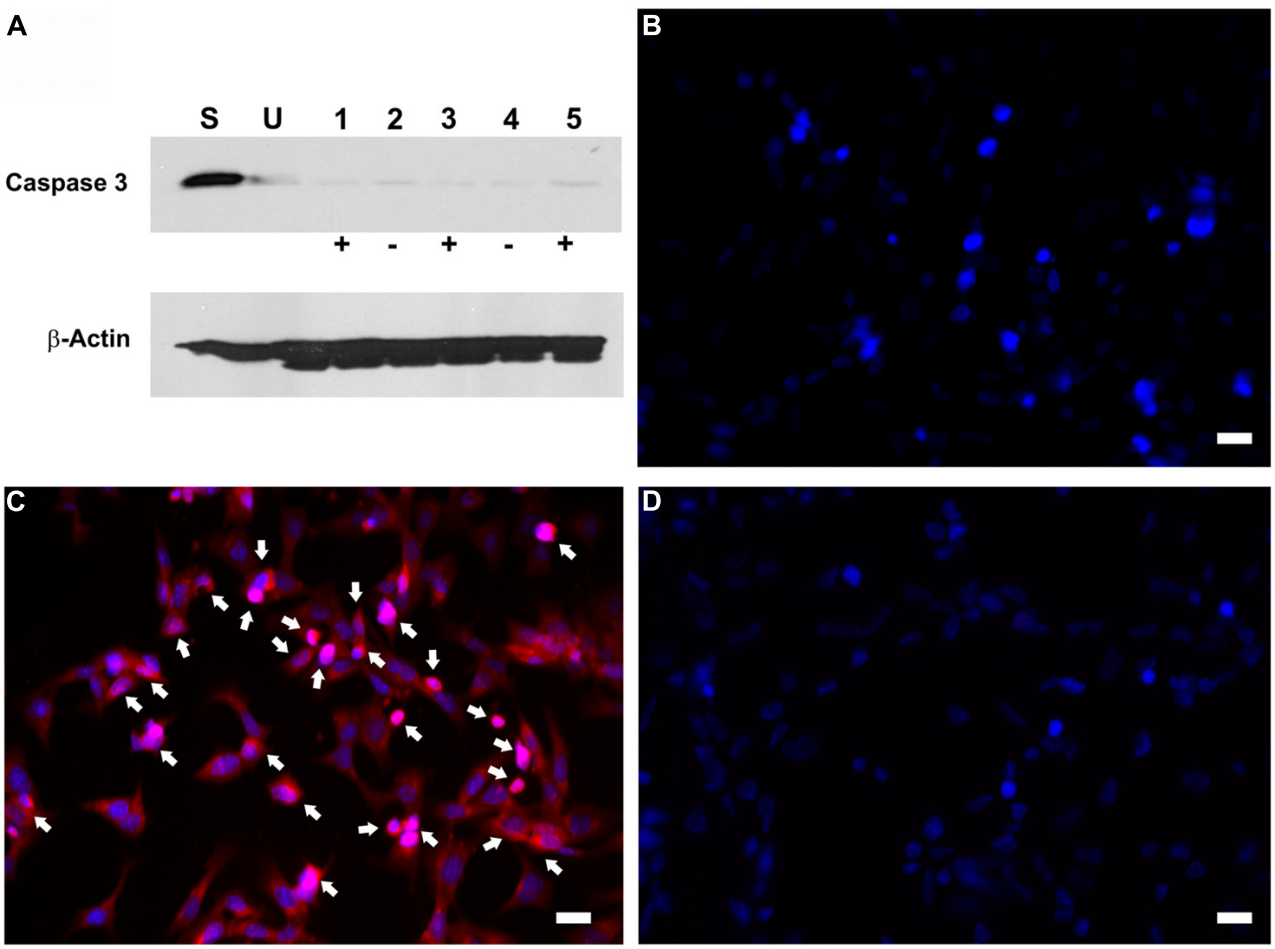
**Dox-mediated DN-CBRb transgene activation has no effect on active caspase 3 activation.** HEI-OCI cells co-transfected with TetO-DN-CB-myc6-Rb1 and the pCMV-Tet3G transactivator vector were cultured in the absence (-) or presence (+) of Dox. **(A)** Protein expression of active caspase 3 in the absence or presence of Dox for 24 and 48 h. **(B–D)** Immunohistochemical analysis of apoptotic cell death by CaspaTag assay. **(B)** Untransfected cells. **(C)** Transfected cells treated with Dox. **(D)** Untransfected cells treated with Staurosporine (positive control). Arrows point to apoptotic cells. S = Untransfected cells treated with Staurosporine for 4 h ; U = Untransfected cells; 1 = Transfected cells treated with Dox only for 48 h; 2 = Transfected cells not treated with Dox for 24 h; 3 = Transfected cells treated with Dox for 24 h; 4 = Transfected cells not treated with Dox for 48 h; 5 = Transfected cells treated with Dox for 48 h. Bar = 10 μm.

To investigate whether the inhibition in RB1 expression leads to increase in cell proliferation, HEI-OC1 cells were co-transfected with pTet-Splice-DN-CBRb and pCMV-Tet3G vectors and submitted or not (control group) to Dox treatment, as described above. Increase in proliferation was quantified by measuring the DNA content in both experimental and control groups, as well as in untransfected HEI-OC1 cells. Consistent with the activation of the DN-CBRb construct, the results demonstrated that co-transfection with pTet-Splice-DN-CBRb and pCMV-Tet3G vectors, followed by Dox-induced gene expression, led to a modest, but significant increase in DNA content compared to the control groups, which is an indirect measure of increase in cell number (**Figure 4**). In contrast, no significant changes in DNA content were observed between transfect HEI-OC1 cells not treated with Dox and untransfected HEI-OC1 (**Figure 4**). Next, to assess the possibility of cell death following activation of the DN-CBRb construct, the protein levels of active caspase 3 were measured by immunoblotting. Positive control consisted of HEI-OC1 cells treated with 1 μM Staurosporine. Overall, no significant changes in active caspase 3 protein levels were observed between untransfected control (treated and untreated with Dox) and experimental (transfected/Dox-untreated and transfected/Dox-treated) cells even after 48 h of transfection (**Figure 5A**). These results were recapitulated by *in situ* caspase detection on untransfected controls and experimental HEI-OC1 cells (**Figures 5B,C**). In sharp contrast, significant cell death was observed on the Staurosporine-treated untransfected HEI-OC1 cells (positive control; **Figure 5D**).

### *IN VIVO* ASSESSMENT OF THE CB-myc6-Rb1 CONSTRUCT ACTIVATION

**FIGURE 6:**
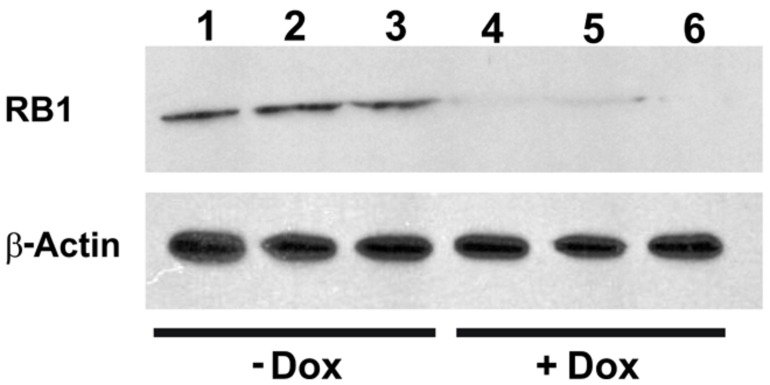
**Dox-mediated DN-CBRb activation is tightly regulated by Dox administration.** Cochleae of Dox treated (lanes 1–3) and untreated (lanes 4–6) P10 DN-CBRb^+^/*ROSA-CAG-rtTA*^+^ mice were submitted to Western blot analysis with anti-RB1 antibody. Consistent with a tight regulation of the DN-CBRb expression by Dox administration, no endogenous RB1 expression was observed on DN-CBRb^+^/*ROSA-CAG-rtTA^+^* mice cochleae in the presence of Dox.

To investigate the functionality of the *CB-myc6-Rb1* construct *in vivo*, the pTet-splice vector containing the DN-CBRb cassette was digested with the *NotI* enzyme to release the transgenic cassette, including the promoter and terminator elements. Gel-purified DNA was used to generate the transgenic mice using standard pronuclear injection techniques. DN-CBRb transgenic lines were further bred to the *ROSA-CAG-rtTA* tetracycline inducer line, which enables ubiquitous expression of DN-CBRb upon Dox administration. Litters from mating of DN-CBRb to *ROSA-CAG-rtTA* mice were genotyped as described in material and methods. DN-CBRb^+^/*ROSA-CAG-rtTA*^+^ and DN-CBRb^-^/*ROSA-CAG-rtTA*^+^ were Dox-induced and analyzed for transgene expression. Ten independent DN-CBRb founder lines were identified. Five of these lines were confirmed for germline transmission of the transgene and used as founders for further breeding. Currently four of those lines are cryopreserved. The results presented here correspond to one single line of DN-CBRb mouse. To examine the *in vivo* feasibility of the method, we bred the DN-CBRb line with the tetracycline inducer *ROSA-CAG-rtTA* mice. DN-CBRb^+^/*ROSA-CAG-rtTA*^+^ mice did not exhibit any deficits and were further used for transgene induction experiments. Furthermore, to test for any potential leaky expression of the DN-CBRb transgene, we measured RB1 protein expression in DN-CBRb^+^/*ROSA-CAG-rtTA*^+^ mice in the absence or presence of Dox. Supporting a tight regulation of the transgene by Dox, a significant decrease in RB1 protein expression was observed in DN-CBRb^+^/*ROSA-CAG-rtTA^+^* mice fed with Dox, but not in the untreated DN-CBRb^+^/*ROSA-CAG-rtTA^+^* mice (**Figure 6**). Given the evidences of specific Dox regulation of DN-CBRb^+^/*ROSA-CAG-rtTA^+^*, added to the fact that no leaky transgene expression was observed on any other genotype combination, either in the presence or absence of Dox, we chose to use DN-CBRb^-^/*ROSA-CAG-rtTA^+^* (western blotting and histology) and DN-CBRb^+^/*ROSA-CAG-rtTA^-^* (RT-PCR) as negative controls.

**FIGURE 7:**
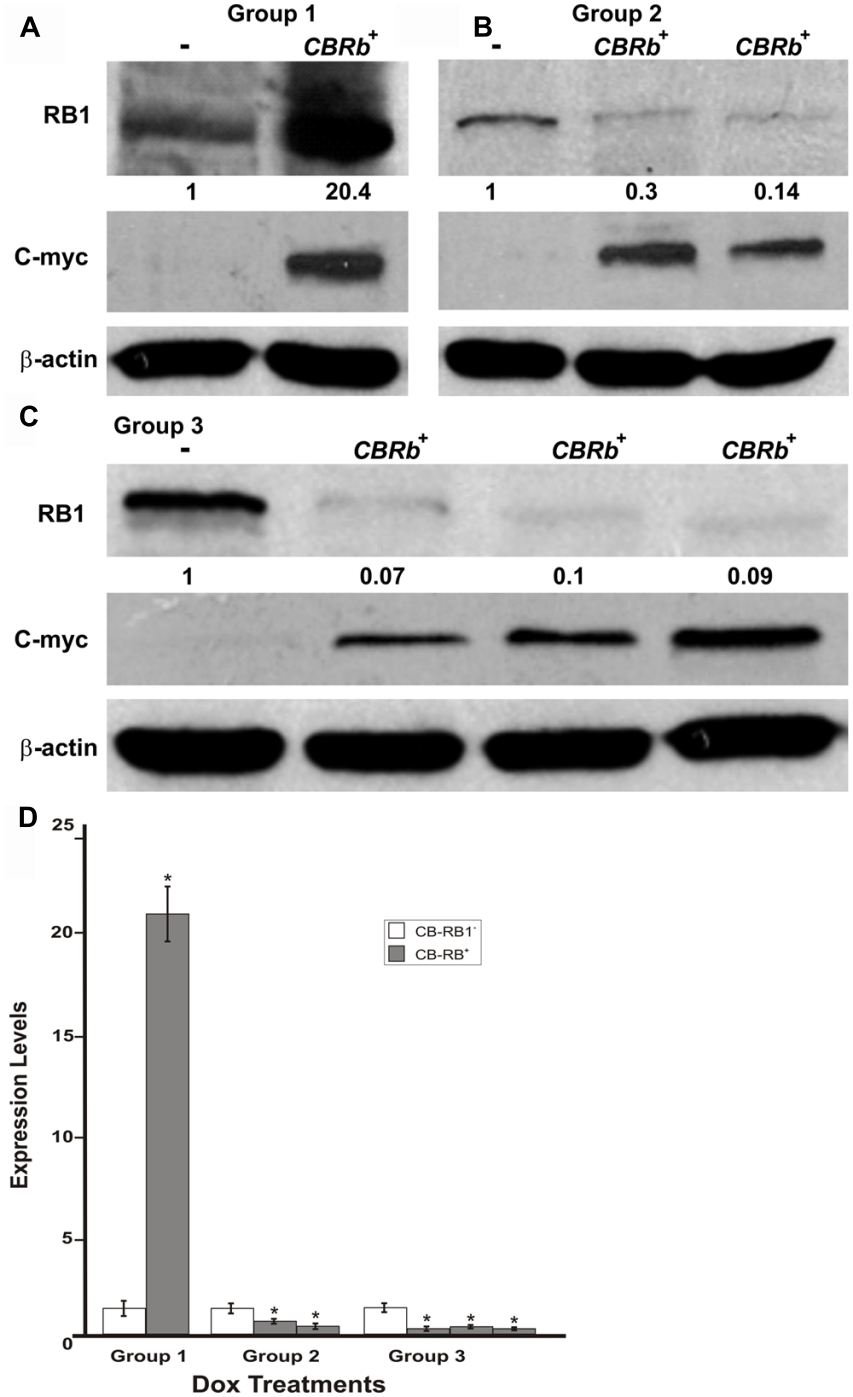
**DN-CBRb transgene activation in the inner ear of postnatal (P) 36 DN-CBRb^+^/*ROSA-CAG-rtTA*^+^ (CBRb^+^) and DN-CBRb^-^/*ROSA-CAG-rtTA*^+^ (CBRb^-^) mice after 3 (group 1), 7 (group 2), and 10 (group 3) days of Dox treatment. (A–C)** Anti-RB1, which reacts with both hyperphosphorylated and hypophosphorylated forms of RB1, as well as anti-c-myc antibodies were used for detection. Densitometric analysis was done using ImageJ software (http://imagej.nih.gov/ij/). The corresponding values obtained were normalized to the negative control CBRb (-) and then to β-actin. The relative RB1 expression levels are shown underneath each blot. **(D)** Graphic representation of normalized RB1 expression levels plotted against the different treatments highlights a consistently lower RB1 detection in CBRb^+^ samples. Each lane on the Western blot gel **(A–C)** and each column on the graphic **(D)** correspond to a different individual and sample. (-) = CBRb^-^; *P < 0.05.

To determine the length of time needed to activate the *ROSA-CAG-rtTA* inducer line and elicit the DN-CBRb activation, DN-CBRb^+^/*ROSA-CAG-rtTA*^+^ mice and DN-CBRb^-^/*ROSA-CAG-rtTA*^+^ mice were divided into three different groups and administered Dox in drinking water for 3 (Group 1), 7 (Group 2), and 10 (Group 3) consecutive days. Immediately after treatment, protein was extracted from postnatal (P) day 36 mice cochleae and analyzed by western blot using mouse anti-RB1 and c-myc hexameric antibodies (**Figures 7A–C**). Consistent with an initial accumulation of RB1 protein, quantification of RB1-Dox dose kinetics revealed a more than 20-fold increase in RB1 protein in cochleae of double-positive mice compared to the negative control samples (**Figures 7A–D**). Such accumulation may reflect the detection of both DN-CBRb and native RB1 subunits, prior to their association and lysosomal degradation. This notion was further supported by the observation that DN-CBRb^+^/*ROSA-CAG-rtTA^+^* cochleae from groups 2 and 3 displayed steadily less RB protein than the control samples (**Figure 7B,D**). Analyses of liver and heart biopsies confirmed the pattern of RB1-Dox dose kinetics described above (data not shown). Of note, no significant differences in RB1 expression were observed in mice treated with Dox for periods longer than 10 consecutive days (data not shown). Hence, all treatments in subsequent experiments were performed on a 10-day Dox treatment.

**FIGURE 8:**
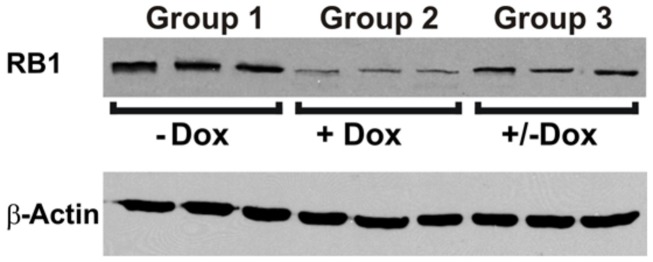
**Reversible *in vivo* DN-CBRb transgene regulation.** P10 DN-CBRb^+^/*ROSA-CAG-rtTA*^+^ mice were fed with Dox in drinking water for 10 consecutive days. Untreated animals of the same genotype were used as control group (Group 1; -Dox). At the end of Dox treatment animals were euthanized either immediately (Group 2; +Dox) or 5 days after Dox withdraw (Group 3; ±Dox). Supporting the reversibility of the system, RB1 levels were visibly decreased on group 2. Nevertheless, cochleae collected 5 days after Dox withdraw showed RB1 levels, which were comparable to those of the control group.

To investigate the reversibility of the DN-CBRb transgene *in vivo*, P10 DN-CBRb^+^/*ROSA-CAG-rtTA*^+^ mice were fed with Dox in drinking water for 10 consecutive days. Controls consisted of untreated DN-CBRb^+^/*ROSA-CAG-rtTA*^+^ mice (Group 1; -Dox). Following that period, animals were euthanized either immediately (Group 2; +Dox) or 5 days after Dox withdraw (Group 3; ±Dox). Cochleae were dissected and analyzed for RB1 expression (**Figure 8**). Compared to the control untreated group, RB1 expression was visibly downregulated on group 1 (**Figure 8**). On another hand, supporting the reversibility of the system, RB1 expression on group 3 was increased compared to group 2. These results are in agreement with previously shown *in vitro* analysis (**Figure 3**).

**FIGURE 9:**
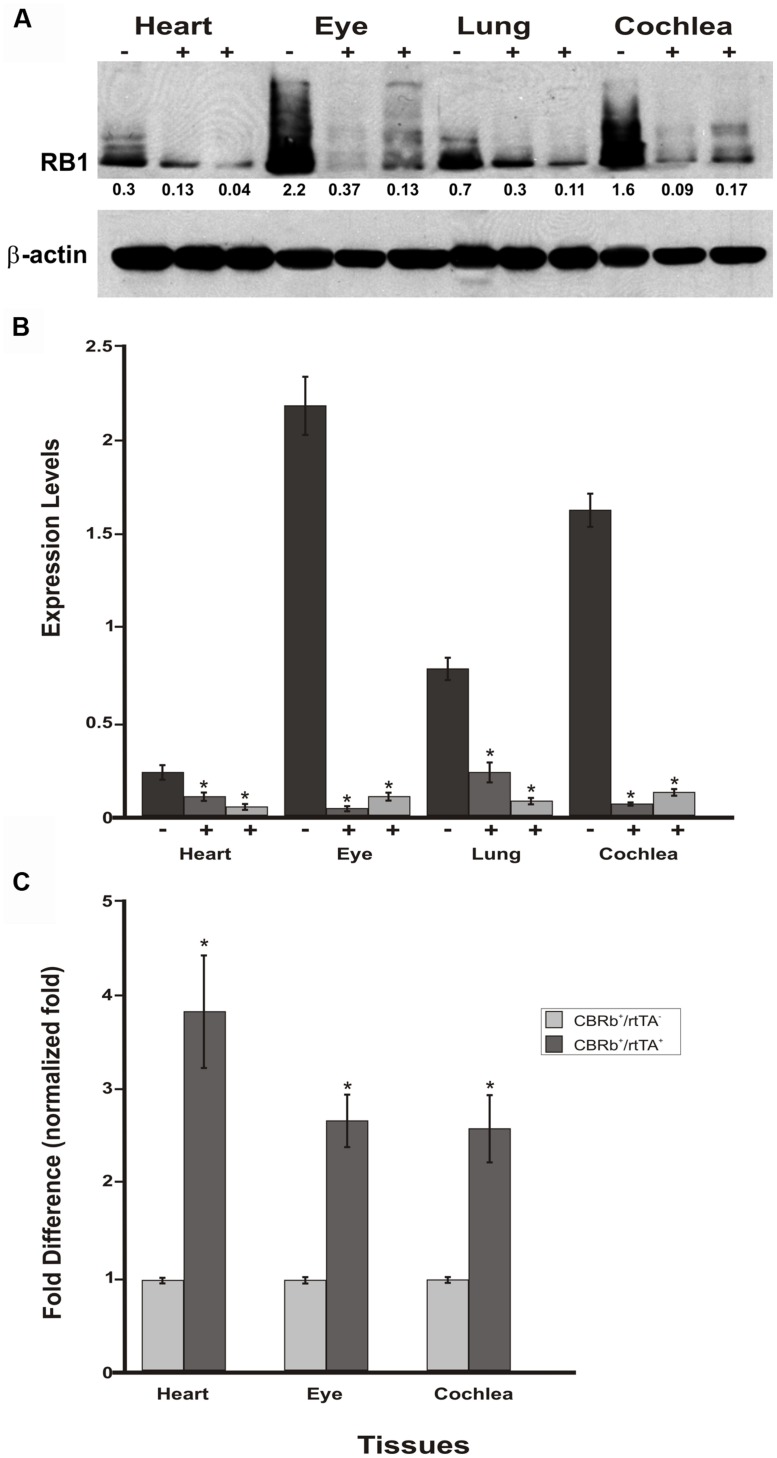
**Spatial analyses of the DN-CBRb transgene activation in P18 DN-CBRb^+^/*ROSA-CAG-rtTA*^+^ and DN-CBRb^+^/*ROSA-CAG-rtTA^-^* mice heart, eye, lungs, and cochlea. (A)** Confirming efficiency of Dox-mediated transgene activation, expression of the endogenous RB1 was downregulated in the CBRb^+^/*ROSA-CAG-rtTA*^+^ (+) tissues, but not in the CBRb^-^/*ROSA-CAG-rtTA*^+^ (-) control mice tissues. Densitometric analysis was performed as described in **Figure 4**. The relative RB1 expression levels are shown underneath each blot. **(B)** Graphic representation of normalized RB1 expression levels plotted against the different tissues. Inter-individual variation on endogenous RB1 levels may explain differences in individual responses to transgene activation and RB1 downregulation. **(C)** RT-PCR analyses in eye, heart and cochleae revealed significant upregulation of the DN-CBRb transcript in dox-treated DN-CBRb^+^/*ROSA-CAG-rtTA*^+^ tissues, but not in DN-CBRb^+^/*ROSA-CAG-rtTA^-^*. CBRb^+^/rtTA^+^ = DN-CBRb^+^/*ROSA-CAG-rtTA*^+^; CBRb^-^/rtTA^+^ = DN-CBRb^-^/*ROSA-CAG-rtTA*^+^; CBRb^+^/rtTA^-^ = DN-CBRb^+^/*ROSA-CAG-rtTA^-^*. *P < 0.05.

To assess any potential tissue-specific variation on the efficiency of the Dox-mediated DN-CBRb activation, DN-CBRb^+^/*ROSA-CAG-rtTA*^+^ and age-matched DN-CBRb^+^/*ROSA-CAG-rtTA*^-^ mice were treated with Dox for 10 consecutive days. Cochleae, lungs, heart, and eye biopsies were dissected from experimental and control groups at P18 and subjected to western blotting with antibody against RB1 (**Figure 9A**). As expected, an inter-tissue variation on RB1 expression levels was observed in the DN-CBRb^+^/*ROSA-CAG-rtTA*^-^ group, with retina and cochleae showing higher RB1 concentrations (**Figure 9A**). Consistent with the notion of a possible interaction between the wild-type RB1 and the DN-CBRb transgene, endogenous RB1 expression was consistently decreased in every one of the DN-CBRb^+^/*ROSA-CAG-rtTA*^+^ mice tissues analyzed (**Figure 8A**). In spite of some inter-individual variations on the residual amount of RB1 protein after transgene activation, RB1 expression was visibly and significantly downregulated compared to the control animals (**Figure 8A,B**). These results were in agreement with our quantitative RT-PCR analyses in eye, heart and cochleae of Dox-treated DN-CBRb^+^/*ROSA-CAG-rtTA*^+^ and age-matched Dox-treated DN-CBRb^+^/*ROSA-CAG-rtTA*^-^ mice, which showed significant upregulation of the DN-CBRb transcript in DN-CBRb^+^/*ROSA-CAG-rtTA*^+^ tissues, but not in DN-CBRb^+^/*ROSA-CAG-rtTA*^-^ (**Figure 9C**). Moreover, RT-PCR analyses not only confirmed the inducibility of the system, by showing no transgene upregulation in the DN-CBRb^+^/*ROSA-CAG-rtTA*^-^ mice, but also demonstrated an increased concentration of the transgene in CBRb^+^/*ROSA-CAG- rtTA*^+^ animals.

Endogenous *Rb1* expression is subjected to temporal and spatial variations (Hamel et al., 1993; Moore et al., 1996; Mantela et al., 2005). The present results collected from four different tissues from P18 mice combined with our previous set of experiments (**Figure 7A–D**) on P36 mice cochleae, support both the temporal and spatial efficiency of the DN-CBRb transgene activation, as well as the utilization of the DN-CBRb mouse model in a variety of studies looking into manipulating RB1 at different postnatal time points.

### DN-CBRb EXPRESSION IN THE MOUSE COCHLEA

**FIGURE 10:**
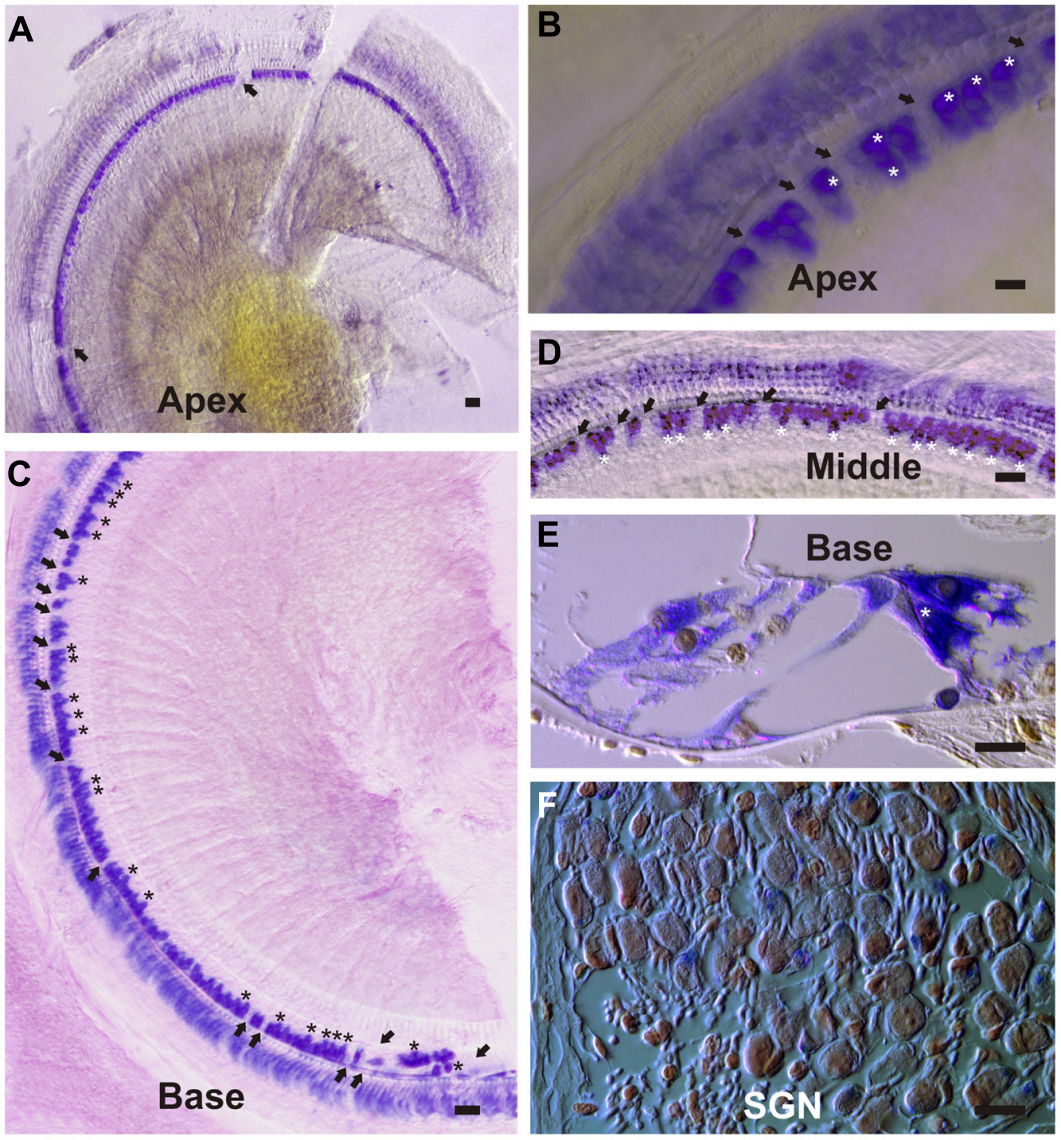
**DN-CBRb transgene expression in a P21 DN-CBRb^+^/*ROSA-CAG-rtTA*^+^ mouse cochlea. (A–F)** Strong cytoplasmic and nuclear ISH signal was observed at the inner hair cells (IHCs), region of Dox-treated DN-CBRb^+^/*ROSA-CAG-rtTA*^+^ mouse cochlea. **(A–D)** Transgene expression was detected throughout the length of the cochlea, but visibly stronger at the middle and basal regions compared to the apex of the cochlea. Mosaic DN-CBRb expression was observed (arrows) and may indicate incomplete recombination in those cells. Likewise, supernumerary cells (asterisk) showed strong hybridization signal, suggesting efficient transgene transmission to the newly formed cells. **(E)** Sections of the cochlea counterstained with Nuclear Fast Red, confirmed our observations on whole mount preparations and allowed for a better appreciation of a supernumerary IHC (asterisk), as well as some light transgene expression in the spiral ganglion neurons **(F)**. SGN , spiral ganglion neurons; Bar = 10 μm.

To investigate cell-specific expression of the DN-CBRb transgene, Dox-treated and untreated (control) P21 DN-CBRb^+^/*ROSA-CAG-rtTA*^+^ mice cochleae were submitted to ISH with transgene-specific riboprobes. Supporting efficient activation and tight regulation of the transgene, strong cytoplasmic and nuclear signal was detected at the IHCs region, including inner pillar cells and border cells, throughout the length of the cochlea (**Figure 10A–E**). Likewise, hybridization signal was observed on supernumerary cells along the length of the cochlea (**Figure 10B,C–E**), suggesting efficient transmission of the transgene to the daughter cells. No hybridization signal was observed on the control samples (data not shown). Light hybridization signal was also observed in the cytoplasma of outer HCs, Deiters’ cells, outer pillar cells, and spiral ganglion neurons (**Figure 10D,F**). Cytoplasmic detection of the transgene further evidences efficient transport of the DN-CBRb mRNA from the cells’ nuclei to be translated into active proteins. Noteworthy, mosaic transgene expression, which is part of the intrinsic nature of transgenic constructs, was also observed on DN-CBRb expression (**Figure 10A–F**). Such phenomenon is likely the result of incomplete recombination, a common occurrence associated with transgenic constructs. Therefore, incomplete recombination associated with the inherent mosaic expression of rtTA inducible systems may explain, at least in part, the patchy pattern of supernumerary cells observed on DN-CBRb^+^/*ROSA-CAG-rtTA*^+^ mouse.

### TEMPORARY *Rb1* DOWNREGULATION IN THE DN-CBRb^+^/*ROSA-CAG-rtTA*^+^ MOUSE RESULTS IN SUPERNUMERARY INNER HAIR CELLS

**FIGURE 11:**
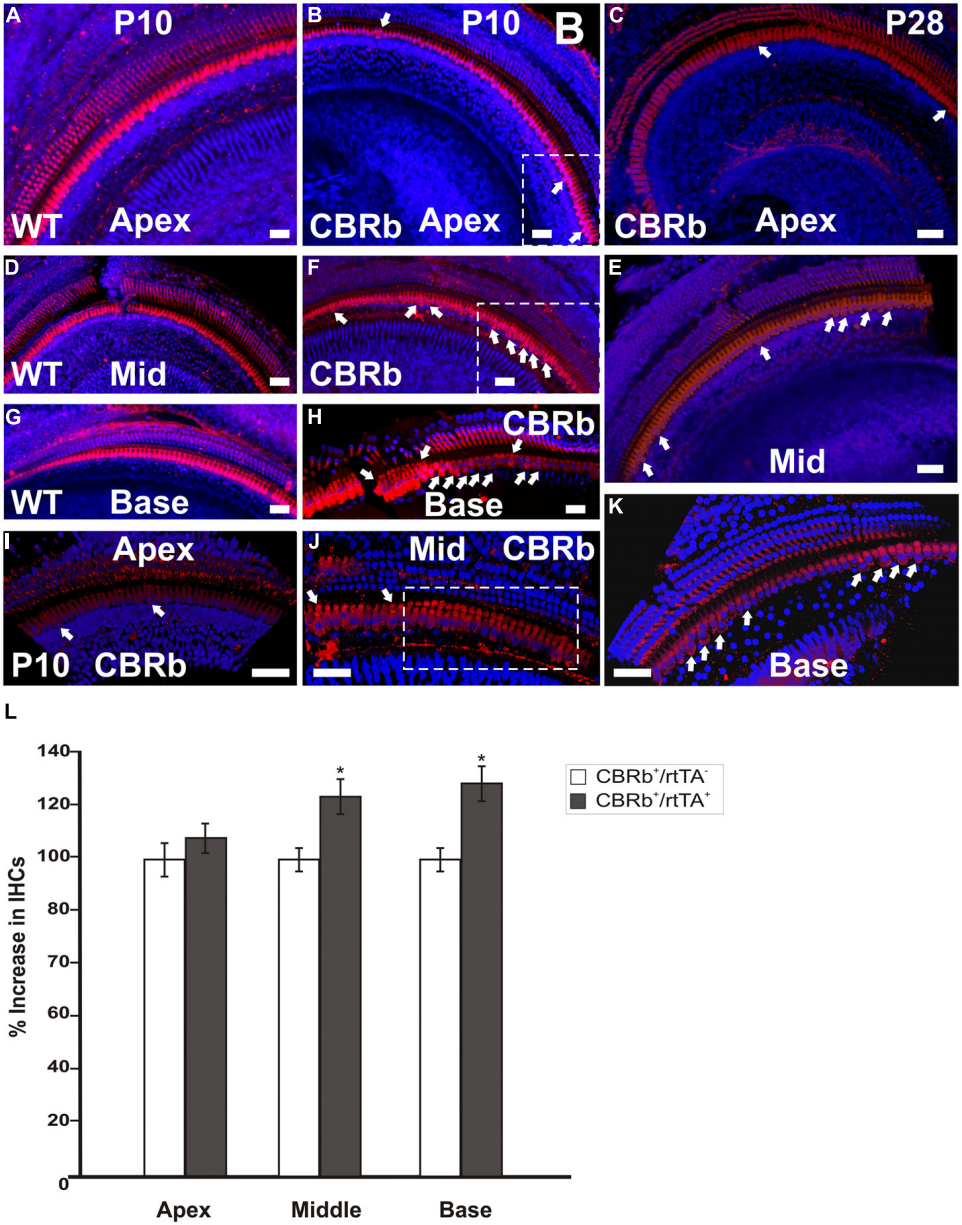
**Immunohistochemical detection of myosin VIIa (M7a)-positive supernumerary cells in the cochleae of DN-CBRb^+^/*ROSA-CAG-rtTA*^+^ (CBRb) at postnatal (P) day 10 and P28.** Compared to the DN-CBRb^+^/*ROSA-CAG-rtTA^-^* (WT) control, which show a single line of cells at the IHCs’ region throughout the length of the cochlea **(A,D,G)**, P10 **(B,E,H–J)** and P28 **(C,F,K)** CBRb mutants exhibited extra M7a-positive cells (arrows and boxed regions), which were more concentrated in the basal and middle turns of the cochlea. Of note, the density of supernumerary cells was higher in P10 ears compared to P28. A close up of boxed areas in **(B,E)** are shown in **(I)** and **(J)**, respectively. Boxed area in **(J)** highlights an area where IHCs are seen in as a double row. The blue background in all figures corresponds to DAPI staining. Bar = 10 μm. **(L)** Counting of Myosin VIIa-positive cells at the IHCs’ region of CBRb mice cochleae at P10 revealed a significant increase in cells, particularly at the middle and basal turns, when compared to DN-CBRb^-^/*ROSA-CAG-rtTA*^+^ mice. Error bars show SEM. *P < 0.05.

**FIGURE 12:**
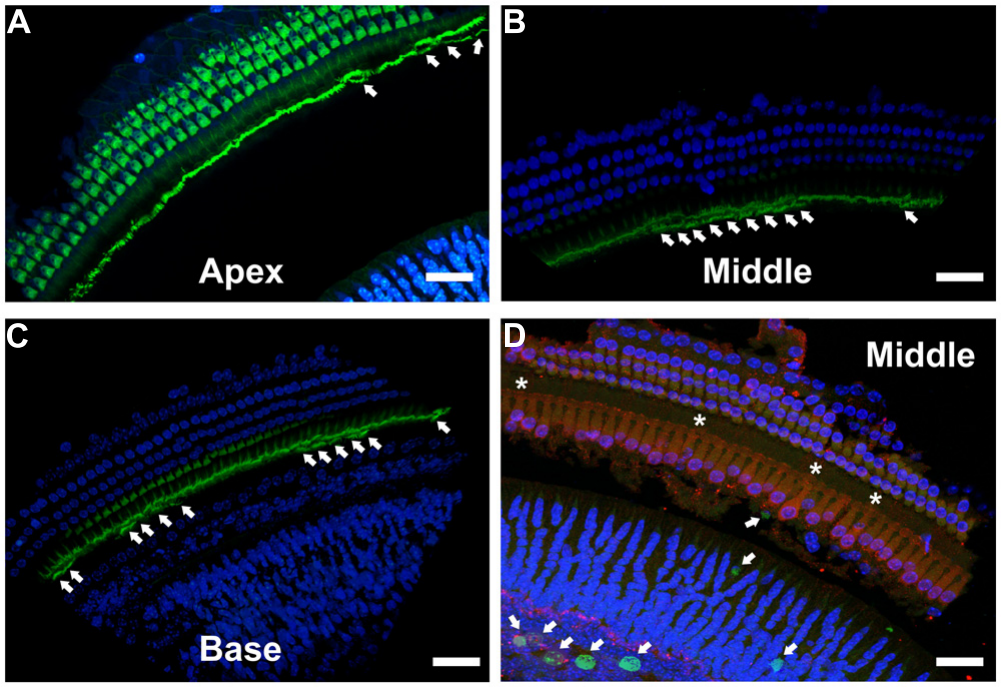
**Immunohistochemical localization of stereocilia and proliferating cells on DN-CBRb^+^/*ROSA-CAG-rtTA*^+^ mouse cochleae. (A–C)** Phalloidin (green) staining of P10 CBRb^+^/*ROSA-CAG-rtTA*^+^ mouse cochlea revealed the presence of F-actin-positive stereocilia-like structures (arrows). Due to the peculiar cochleae anatomy, **(B,C)** were rotated to facilitate visualization of the stereocilia. **(D)** EdU (green) labeling in DN-CBRb^+^/*ROSA-CAG-rtTA*^+^ (CBRb) at P10 showed no proliferation within the OC; however, a few EdU-positive cells were observed at the greater epithelial ridge. Asterisks indicate supernumerary cells. DAPI = Blue; Myosin VIIa = Red. Bar = 10 μm.

To better understand the possible consequences of temporally regulated RB1 inhibition in the inner ear, cochleae of DN-CBRb^+^/*ROSA-CAG-rtTA*^+^ and DN-CBRb^-^/*ROSA-CAG-rtTA*^+^ mice at P10 and P28 were dissected and submitted to immunohistochemistry with antibody against the HC marker Myosin VIIa (M7a). To date, a total of 40 ears (five mice per genotype × 2 time points) have been analyzed. While no changes have been observed in the cochleae of DN-CBRb^-^/*ROSA-CAG-rtTA*^+^ mice, supernumerary M7a-positive cells were observed at the IHCs’ region of DN-CBRb^+^/*ROSA-CAG-rtTA*^+^ cochleae at the two time points analyzed (**Figures 11A–K**). Counting of those supernumerary cells was performed and the results compared between the different turns of the cochlea (i.e., apex, middle, and base) and time points by *t*-test (**Figure 11L**). No statistical differences were observed on the concentration of supernumerary cells between the middle and basal turns at either time points. Nevertheless, comparison between the numbers of supernumerary cells within the combined middle and basal turns and the apical turn was found to be significant (*p* < 0.05). Likewise, DN-CBRb^+^/*ROSA-CAG-rtTA*^+^ mouse at P10 showed significantly more supernumerary cells (*p* < 0.05) than at P28. These results are in agreement with the expression pattern of the endogenous *Rb1* gene in the mouse cochlea (Mantela et al., 2005), showing a base-to-apex expression gradient, which is downregulated during postnatal development. Of note, no significant changes were observed in OHCs. Next, to start gathering information on the morphology of those supernumerary cells, we looked for the presence and morphology of stereocilia by using phalloidin labeling on DN-CBRb^+^/*ROSA-CAG-rtTA*^+^ cochlea at P10. As whole, no visible differences were observed on stereocilia morphology, density, and orientation between supernumerary and regular IHCs at P10 (**Figure 12A–C**). Additional analyses are currently underway to assess functionality and maturation of those newly generated HCs.

Unscheduled proliferation of inner ear cells upon complete or conditional RB1 deletion generally leads to massive cell death (Mantela et al., 2005; Sage et al., 2005, 2006). In the absence of any detectable apoptotic-like features in Dox-treated DN-CBRb^+^/*ROSA-CAG-rtTA*^+^ cochlea, we performed immunohistochemical analysis using antibody specific to detect any potential active caspase 3 activity in those mice cochleae at P10. Cochleae treated with Staurosporine were used as a positive control. No positive caspase 3 labeling was observed, except for a single positive nucleus at the basal turn of one of the ten cochleae analyzed (data not shown). Likewise, EdU labeling of DN-CBRb^+^/*ROSA-CAG-rtTA*^+^ cochlea did not reveal any detectable signs of proliferation within the OC (**Figure 12D**). However EdU-positive cells were still observed in the greater epithelial ridge (**Figure 12D**), suggesting that proliferation of the cells giving rise to the supernumerary HCs took place earlier during the 10 days of Dox treatments. Additional analyses of DN-CBRb^+^/*ROSA-CAG-rtTA*^+^ mice cochleae submitted to shorter periods of Dox treatment is likely to shed light on the timing when proliferation is taking place. No EdU-positive cells were observed in the control DN-CBRb^-^/*ROSA-CAG-rtTA*^+^ mice ears (data not shown).

## DISCUSSION

While the use of *Rb1* knockout (KO) mice has highlighted *Rb1*’s potential role in HC regeneration, early embryonic lethality of *Rb1* KO mice poses serious limitations to undertaking any further regeneration studies *in vivo*, particularly in the late embryonic and postnatal stages (Lee et al., 1992; Wu et al., 2003; Mantela et al., 2005; Sage et al., 2005, 2006). Moreover, conditional *Rb1* deletion in specific tissues using the Cre/lox system is not always straightforward (Weber et al., 2008; Turlo et al., 2010). To circumvent the problems associated with such approaches, we sought to generate a mouse model in which RB1 can be conditionally inactivated by overexpressing a DN mutant RB1 in a spatiotemporal manner. To abolish the function of endogenous RB1, a number of options have been proposed (Dick, 2007; Macpherson, 2008). The best alternative would be the one that results not only in the inactivation of the endogenous RB1, but also its proteolytic degradation. Such a transgenic construct has been previously described elsewhere (Higashitsuji et al., 2000; Li et al., 2000). However, while elegant, it lacked a mechanism to control transgene activation, response, or tissue specificity. We have generated an inducible RB1 DN transgenic mouse model that combines the straightforward strategy of the Dox-dependent transcriptional system to the fusion of the lysosomal protease CB with *Rb1* to generate a temporally regulated Dox-mediated DN-RB1 mouse model. Unlike other methods for designing a DN mutation, the proposed method requires minimal knowledge of the gene’s structure and function and can be potentially applied to any gene of interest (Li et al., 2000). Moreover, perhaps the most exciting aspect of this method is the reversibility of the inducible expression of the transgene, *in vitro* and *in vivo*, after Dox withdrawn.

*Rb1* is a classical tumor suppressor gene with a variety of functions, ranging from DNA replication to mitosis, DNA repair, DNA damage checkpoint control, cellular senescence, differentiation, to apoptosis (Korenjak and Brehm, 2005). In addition to canonical binding with E2F during the G1 phase and regulating proliferation, RB1 binds to a number of transcription factors via protein–protein interactions to regulate tissue-specific functions (Welch and Wang, 1993; Dick and Dyson, 2003). To our knowledge, there is no evidence of direct physical interaction between RB1 molecules. Nevertheless, we hypothesize that some of the numerous RB1-binding molecules will have multiple RB1 sites, thus allowing for the presence of multiple RB1 molecules at a given time. Consistent with our hypothesis, BRCA1 has been shown to have RB1-binding sites in both the C- and N-terminus (Aprelikova et al., 1999; Yarden and Brody, 1999). In this light, if one of the RB1 molecules is a DN mutant, it will prevent RB1-containing complex from fulfilling their normal role, decreasing the endogenous RB1 levels and eliciting an RB1-null phenotype (Kominami et al., 1991). While the full biochemical mechanisms underlying the DN-CBRb activity are yet to be unveiled, *in vivo* and *in vitro* downregulation of the endogenous RB1 protein following Dox treatment suggests that, like the BRCA1 protein, more members of the RB1 interactome may have multiple RB1-binding sites.

In addition to its well-established importance in development and cancer research, special attention has been paid to the role of *Rb1* in tissue regeneration (Bakay et al., 2006; Wang et al., 2013), particularly auditory HC regeneration (Mantela et al., 2005; Sage et al., 2005, 2006; Weber et al., 2008). During the past 8 years, using the auditory system as a model, we and others have shown that manipulation of the *Rb1* gene or specific components of the *Rb1* pathway trigger unscheduled proliferation of the otherwise mitotically quiescent neurosensory HCs and their clonally related supporting cells (Mantela et al., 2005; Sage et al., 2005; Weber et al., 2008; Rocha-Sanchez et al., 2011, 2013). Although a growing repertoire of molecular techniques has substantially contributed to our understanding of the multifaceted role of *Rb1*, no approach to date has been effective in allowing for the understanding of RB1 activity without permanently eliminating its activity, which, given *Rb1*’s broad spectrum of interactions, generally results in either impaired viability or morbidity (Lee et al., 1992; Wu et al., 2003). In regard to tissue regeneration, induced and reversible temporary inactivation of RB1 and its associating factors may lead to the expansion of targeted cells without triggering cell death, because RB1 function will be restored once Dox induction is withdrawn.

We have generated a mouse model that allows for inducible, reversible, and temporal control of the RB1 protein inhibition at various postnatal ages. Initial studies on DN-CBRb mice demonstrate proof of concept for the inducible and reversible inhibition of endogenous RB1 expression. In the present study, we have utilized a generic *ROSA-CAG-rtTA* tetracycline inducer line to trigger DN-CBRb expression. *In vivo* and *in vitro* analyses support the effectiveness of this model to induce cell proliferation at rather moderate levels, compared to the conventional *Rb1*-KO mouse model, without trigering apoptosis (Mantela et al., 2005; Sage et al., 2005, 2006; Weber et al., 2008). Contrasting with the previously described *Rb1* mouse models, residual RB1 protein expression is still observed in the DN-CBRb^+^/*ROSA-CAG-rtTA*^+^. Such RB1 expression levels seem to be low enough to release those quiescent cells from their postmitotic arrest, but not to trigger apoptosis, as assumed from our present results. At this point of our study, we have no experimental evidences of apoptotic cell death following Dox-mediated DN-CBRb activation either *in vitro* or *in vivo*. An equally significant finding was the observation of supernumerary M7a cells in the DN-CBRb^+^/*ROSA-CAG-rtTA*^+^ mouse cochleae at two different postnatal time points. While the number of supernumerary cells was higher at P10 than at P28, it is well established in the auditory field that cell proliferation does not naturally occur once the cells have exit the cell cycle, which happens during embryonic development. Hence, even the smaller number of supernumerary cells at P28 is still a positive finding. Moreover, attesting to the specificity of the transgene activity and consistent with the *Rb1* expression pattern in the postnatal cochlea (Mantela et al., 2005), transgene expression and the resultant supernumerary cells seems to follow a base-to-apex gradient of concentration, which is most noticeable at the IHCs’ region, where *Rb1* expression has been previously described (Mantela et al., 2005). As a matter of fact, all evidences collected so far, suggest that supernumerary cells in the DN-CBRb^+^/*ROSA-CAG-rtTA*^+^ mouse cochleae may have been originated from proliferation of those cells showing upregulation of the DN-CBRb transgene, particularly the inner IHCs’ associated supporting cells.

The selective suppression of gene expression is both an experimental tool for defining function and a potential means to medical therapy. Although our research is focused in the auditory system, in view of the central role played by RB1 in various disease process and lack of available animal models to modulate its activity, the DN-CBRb model should be a valuable resource to researchers across various fields of study. While the molecular etiology of the retinoblastoma eye tumor is one of the simplest among all human cancers (Goodrich, 2006), the significance of RB1 functional complexity and interactions, as well as its relevance in normal development and malignancies, is a work in progress. For example, germline mutations in RB1 predispose an individual to a very limited set of cancers (Kovesdi et al., 1987; Roarty et al., 1988). In contrast, somatic mutations in RB1 or in members of its interactome appear to contribute to malignancy in a wide variety of tissues. This apparent time-dependency on *Rb1* function only widens the current gap between molecular studies of *Rb1*-mediated gene regulation and the resultant phenotypes *in vivo*. While *Rb1* is inactivated in the majority of the malignancies, some types of cancers, such as colon cancer, require constitutive RB1 expression to maintain proliferation and prevent apoptosis (Ali et al., 1993; Du and Searle, 2009; Collard et al., 2012). In such scenarios, temporary inhibition of the RB1 function can potentially lead to the killing of cancer cells or an increase in their sensitivity to radio- or chemotherapeutic agents (Knudsen and Knudsen, 2008; Du and Searle, 2009). On the other hand, in the auditory system, transiently controlled RB1 downregulation may offer an alternative to drive mitotically quiescent cells back into the cell cycle as a mean to regenerate lost sensory HCs.

In summary, we have generated a novel inducible, regulated, and reversible DN-CBRb mouse model, which is the first animal model to achieving a DN inhibition of RB1 protein. To test the efficiency of the construct and potential of the DN-CBRb mouse model we bred it to a generic rtTA mouse line. However, to achieve tissue-specific RB1 inhibition, the DN-CBRb mice can be bred to any other inducer line, making it a suitable model for a variety of different studies in the auditory system and elsewhere.

## Conflict of Interest Statement

The authors declare that the research was conducted in the absence of any commercial or financial relationships that could be construed as a potential conflict of interest.
